# Novel distribution pattern between coexisting sexual and obligate asexual variants of the true estuarine macroalga *Ulva prolifera* †

**DOI:** 10.1002/ece3.2149

**Published:** 2016-04-27

**Authors:** Masanori Hiraoka, Motoki Higa

**Affiliations:** ^1^ Usa Marine Biological Institute Kochi University 194 Inoshiri, Usa Tosa Kochi 781‐1164 Japan; ^2^ Laboratory of Plant Ecology Faculty of Science Kochi University 2‐5‐1 Akebono‐cho Kochi 780‐8520 Japan

**Keywords:** Asexual, coexistence, frozen niche variation model, river estuary, sexual, *Ulva prolifera*

## Abstract

Where sexual and asexual forms coexist within a species, the asexuals are often found to be prevalent in marginal habitats. This asexual distribution pattern has received evolutionary attention linked to the paradox of sex. In many marine species, there is a distributional trend of asexual modes being more common in lower salinity waters regarded as the ecogeographic marginal, being explained by negative effects of low salinities on sexual reproductive success. However, the distribution pattern of estuarine species recently adapted to low salinity waters has remained unknown. The brackish macroalga *Ulva prolifera* being a major benthic component of estuarine ecosystems includes a sexual variant and obligate asexual variants by means of motile spores. We examined the sexual–asexual distribution pattern of this alga along a salinity gradient in river estuaries. Surprisingly, opposite to the distributional trend of marine organisms, the results clearly showed the persistent predominance of sexuals in the lower salinity reaches than the asexuals. In addition, a frequent alternating of dioecious gametophytes and sporophytes in the sexual population was observed, suggesting the sexual reproductive process would be robustly performed in the low salinity waters. Considering *U. prolifera* had evolved from the ancestral marine species to become a true estuarine species of which the core habitat is the low salinity reaches, in a broad sense its sexual–asexual distribution pattern would be involved in asexual marginal occupations of the species range previously reported in other organisms. Based on the frozen niche variation model, we can give a concise explanation for the evolutionary process of this pattern; multiple asexuals with frozen genotypic variation had arisen from sexual ancestors undergoing low salinity adaptation and share the estuarine habitat with the sexuals at present.

## Introduction

The coexistence of closely related sexuals and asexuals is thought to be a fundamental paradox and has received attention from ecologists and evolutionary biologists (Spitzer and Haygood 2007; Silvertown 2008; Lehto and Haag 2010; Gilabert et al. 2014). Except the reproductive mode, such asexuals can be ecologically similar to their sexual progenitors, making a stable coexistence with those progenitors theoretically difficult (Spitzer and Haygood 2007). Evolutionary theory predicts that competition for the same ecological niches leads to the exclusion of one or to their partition (Case and Taper 1986; Peck et al. 1998). Therefore, a comparative study of actual populations sympatrically including sexual and asexual variants must give an important clue to resolve the paradox. However, Kobayashi et al. (2013) mentioned that such a comparison is almost impossible because in most organisms, the asexual form is not found or only seen temporarily, and in a few species where sexual and asexual variants coexist, the asexuals are not usually isolated genetically. An asexual lineage genetically isolated from the sympatric sexual progenitors is valuable for a direct comparison.

**Figure 1:**
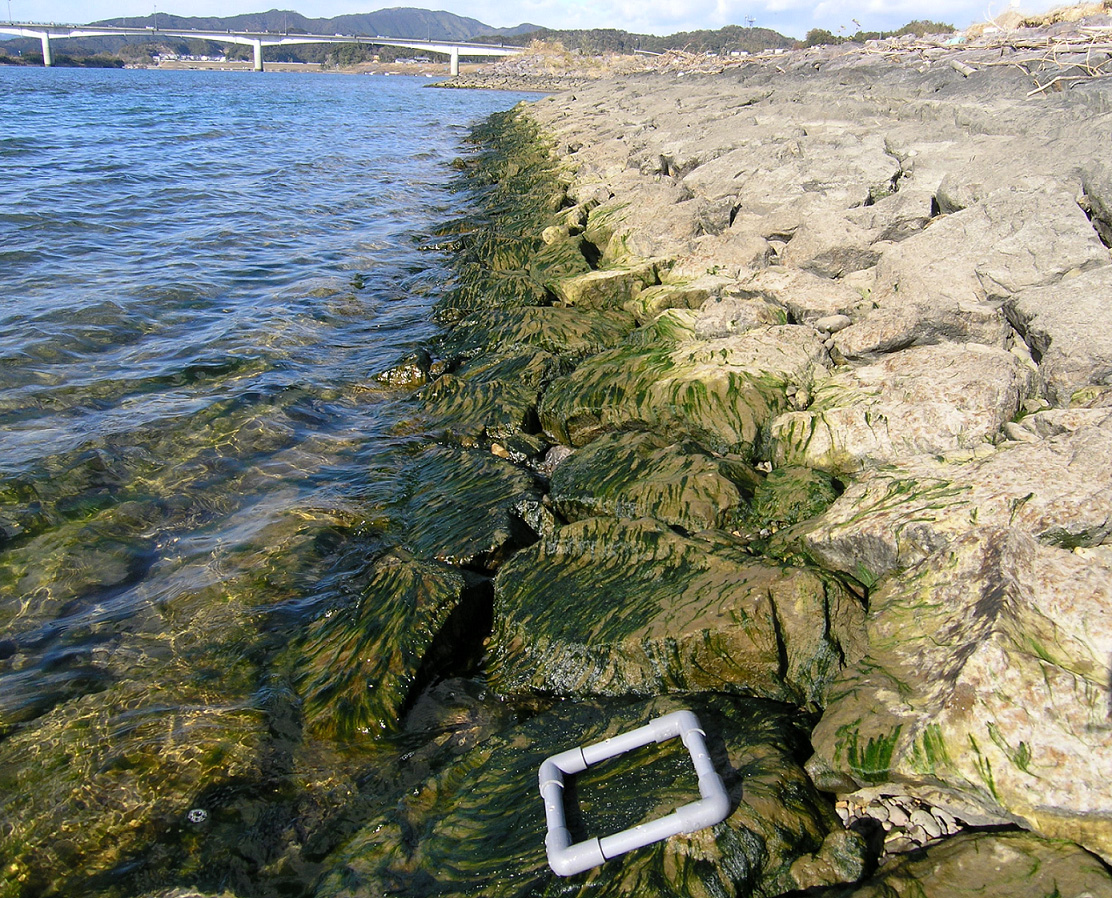
*Ulva* belt along the bank at the lowest tide in the Shimanto River estuary. Quadrat is 15 × 15 cm in size.

The green macroalga *Ulva* is a good candidate for such a comparative study, because it includes their sexual variants and genetically separated asexual variants. Cold–temperate river estuaries have become commonly covered in profuse growths of *Ulva*, mainly *Ulva prolifera* (syn. *Enteromorpha prolifera*), developing as mats and being a major benthic component of the estuarine ecosystem (McLusky and Elliott 2004; Fig. 1). The brackish *U. prolifera* in estuaries in East Asian countries has been utilized as an important commercial food resource (Ohno 1993; Park and Hwang 2011; Liu et al. 2013). The Shimanto River estuary on Shikoku Island, southwestern Japan, is the best known site for the largest harvest of the wild fronds (Ohno and Takahashi 1988; Ohno et al. 1999). In Shikoku Island, three different types of life history have been found (Hiraoka et al. 2003; Fig. S1). One type is the typical sexual life history of genus *Ulva*, which is isomorphic and diplohaplontic, with alternating dioecious gametophytes producing biflagellate anisogametes and sporophytes producing quadriflagellate meiospores. The other two types are asexual life histories clonally reproducing through biflagellate or quadriflagellate diploid zoids specialized for settlement. These asexual zoids were termed “zoosporoids” by Bliding (1948, 1963). A recent study demonstrated that these two asexual variants show high levels of heterozygosity and are suggested to be originally created from hybridization between genetically distinct gametophytes (Ogawa et al. 2014). However, the distribution pattern between sexual and obligate asexual variants in the *Ulva* populations remained unknown. That is partly because a total of five types of the thallus individuals in *Ulva* are not distinguished by morphology even at microscopic level. Furthermore, no appropriate molecular marker to identify each generation or life‐history type has yet been found, although some DNA sequences such as the nuclear encoded rDNA internal transcribed spacer (ITS) region or the 5S rDNA spacer region used for phylogenetic analysis at the intraspecific level were examined (Hiraoka et al. 2003, 2011). In order to identify the reproductive mode of each thallus individual, we must directly look into which type of zoids it releases. Therefore, the “punching method” has been improved for the artificial induction of reproductive maturation and release of zoids from unidentified *Ulva* thalli within a few days (Hiraoka and Enomoto 1998; Dan et al. 2002). Using this method, population dynamics of the five types of the *Ulva* thallus can be revealed.

**Figure 2:**
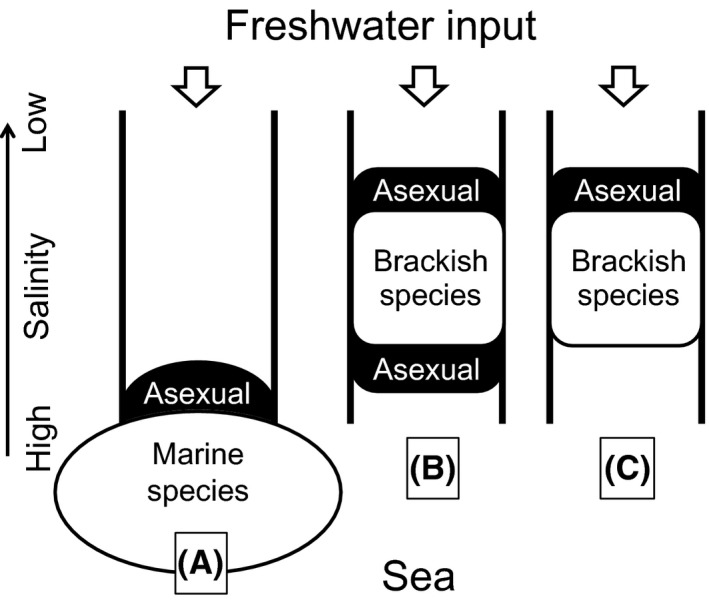
Asexual distribution patterns in the margins of the species range along a salinity gradient in river estuaries. (A) Previously reported pattern in many marine species. (B) An upper and lower marginal dominant pattern for brackish species simply expected by marginal adaptation theories. (C) An upper marginal dominant pattern for brackish species expected from (A) of examples in marine species.

Asexual organisms in animals and plants often have larger distributions and occur at higher elevations and at higher latitudes, and colonize more frequently previously glaciated or devastated areas than their sexual relatives, and these distribution patterns have been called geographical parthenogenesis (Hörandl 2009; Cosendai et al. 2013). Such environments where the asexuals occur are summarized as marginal habitats of the species range (Haag and Ebert 2004; Kawecki 2008; Vrijenhoek and Parker 2009). This pattern has been reported also in marine organisms and particularly well documented in the Baltic Sea which is one of the largest brackish bodies of water in the world. The Baltic Sea is regarded as geographically and ecologically marginal due to the harsh physical conditions for marine species with permanently low salinities (Johannesson and André 2006). A number of marine vascular plant and algal species have a distributional trend of asexual modes being more common inside than outside the Baltic Sea (Reusch et al. 2000; Gabrielsen et al. 2002; Tatarenkov et al. 2005) as shown in Figure 2A. The asexual dominance in the lower salinity regions is explained by negative effects of low salinities on sexual reproductive success and consequently selective advantage of the asexuals (Gabrielsen et al. 2002; Tatarenkov et al. 2005). Considering a brackish species in river estuaries, because its core habitat is the low salinity reaches, two sexual–asexual distribution patterns can be hypothesized according to the previous studies; one is a simple asexual dominance in both upper and lower margins (Fig. 2B), and another is an asexual dominance in the lower salinity margin like marine species (Fig. 2C).

The objectives of this study were (1) to examine whether the estuarine species including sexual and obligate asexual variants shows the asexual marginal distribution pattern previously reported in other organisms, especially in marine species and (2) to understand the reproductive structure of *Ulva* populations, focusing on the largest Japanese *U. prolifera* population in the Shimanto River estuary and two other populations on Shikoku Island. Furthermore, we attempt to explain its evolutionary process by a hypothesis for the geographical parthenogenesis which has been supported by recent studies. In order to clarify these issues, each reproductive mode and life‐history type of a total of more than six thousands of *Ulva* individuals monthly collected from the river estuaries over about 3 years were identified by culture and hybridization experiments. Here, we reveal a novel sexual–asexual distribution pattern of the ephemeral brackish macroalga.

## Materials and Methods

### Study sites and samplings

**Figure 3:**
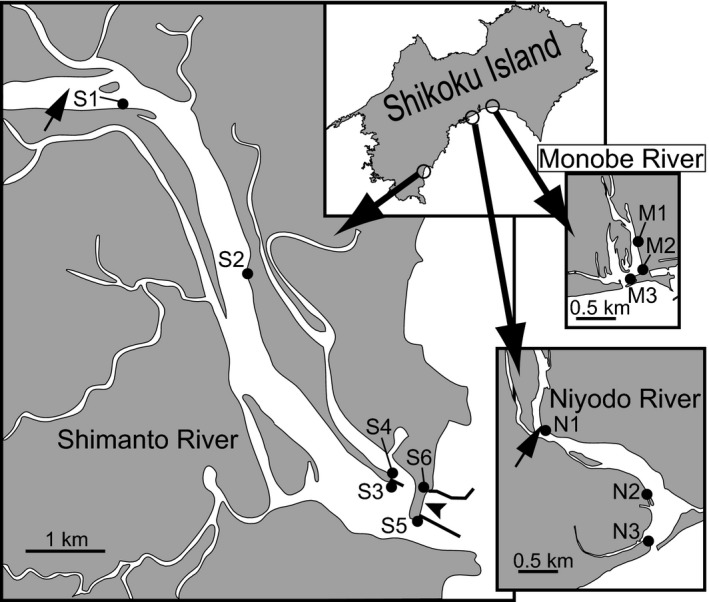
Sampling sites in three river estuaries of Shimanto River (S1‐6), Niyodo River (N1‐3) and Monobe River (M1‐3). Small arrows show upstream limit of *Ulva prolifera* population in the growing season. Arrowhead shows the sandbar, which disappeared in September 2005 and was re‐established in October 2006.

Detailed horizontal and vertical distributions of the *U. prolifera* population have been previously reported in the Shimanto River estuary (Ohno and Takahashi 1988; Ohno et al. 1999). These papers described that the *Ulva* population occurred as a continuous green belt having a vertical width of 1.4–3.5 m on tidal slopes of the estuary from the river mouth to approximately 7 km upstream during the period from the end of October to May. Based on the descriptions, five sites of S1‐5 were selected between the lower and upper limits of the *Ulva* populations as shown in Figure 3. Samplings of *Ulva* thalli were made monthly at around the lowest tide of each month from October 2004 to July 2007. More than 60 *Ulva* thalli with a thin and tubular morphology >1 cm in length were collected randomly and whole widely from the green belt of about 5 m breadth along the bank at each site, as shown in Figure 1; photograph was taken at study site S2 on 18 December 2005. In the middle of the survey period, the sandbar covering more than half of the river mouth disappeared after a typhoon in September 2005, although such a sandbar loss had not happened for the last several decades (Sakaguchi et al. 2009). About 1 year later, the sandbar spontaneously re‐established in October 2006. During the sandbar loss, instead of S5 where it was not possible to approach, the samplings were made at a new site S6 and resumed at S5 after its re‐establishment. Additionally, the same samplings were also conducted at three sites of N1‐3 in the Niyodo River estuary and at three sites of M1‐3 in the Monobe River estuary from October 2005 to July 2007 (Fig. 3). Salinity and water temperature at each sampling time were measured using a salinometer (DIGI‐Auto 3G; Tsurumi Seiki, Yokohama, Japan, or YSI Model 85, YSI Inc., Yellow Springs, OH, USA) and a thermometer (SK‐250; Sato Keiryoki Mfg., Tokyo, Japan).

### Identification of species and reproductive mode

Thallus length of 40–60 intact individuals collected at each site was measured in the laboratory immediately after collection. The thallus of the individual was washed in freshwater to remove sediment and other contaminants and placed separately in a plastic dish (90 mm diameter, 15 mm high), 6‐well, 12‐well, or 24‐well plates filled with autoclaved seawater. The separated *Ulva* individual was microscopically observed for vegetative cells for the species identification based on the previous description of *U. prolifera* (Hiraoka et al. 2003). In order to examine the phase of life histories and sexuality of the individuals, induction of zoid formation was made using the punching method (Dan et al. 2002). Zoid formation was induced within 2–3 days by excision of small thallus fragments (2–5 mm long) from the apical part of the thallus and incubation in 48‐well plates filled with sterile enriched seawater containing 0.05% Porphyran–Conco (Daiichi Seimo, Kumamoto, Japan) which is a nutrient solution for macroalgal culture, at 20°C, 12 h:12 h light:dark cycle (Light period 7:00–19:00) and fluorescent light at 100–150 *μ*mol·photons·m^−2^·s^−1^. The mature fragments released zoids from the morning to noon on the second or third day after the excision. In the same daytime before losing their motile and mating activities, the zoids were observed for microscopic morphology and used for crossing tests. By the observation of flagella, biflagellate zoids were distinguished from quadriflagellate zoids. Furthermore, biflagellate zoosporoids of obligate asexual life history being negative phototactic and 8–9 *μ*m in length were distinguished from biflagellate gametes with intensive positive phototaxis and 6–7 *μ*m in length (Hiraoka et al. 2003). As quadriflagellate zoosporoids of obligate asexual life history (<10 *μ*m length) were smaller in size than quadriflagellate meiospores of sexual life history (>11 *μ*m length) (Hiraoka et al. 2003), the thallus that released unambiguously small quadriflagellate zoids was identified as having an asexual life history. However, when intermediate‐sized quadriflagellate zoids were released in some cases, they were isolated and cultured up to the next generation under the incubation conditions mentioned above. Their life‐history type was determined by type of zoids released from the next generation, biflagellate gametes or the same type of quadriflagellate zoids (cf. Fig. S1). Sexuality of gametophytes was determined by crossing tests. Small droplets of suspended gametes from two gametophytes concentrated by their positive phototaxis were placed on a glass slide and mixed suspension was microscopically observed. On each month's collection, when large gametes and small gametes were first found to conjugate, their mother thalli were identified as female and male gametophytes, respectively. The other gametophytes in the same collection were tested to cross with the identified male and female gametophytes to determine their sexuality.

### Statistical analysis

To compare compositional similarities of sexual and asexual variants of *U. prolifera* and other *Ulva* species among the study sites and among the growing seasons in the estuaries of the Shimanto, Niyodo, and Monobe rivers, cluster analysis was conducted. Mean numbers of each element (i.e., sexual and asexual variants of *U. prolifera* and other *Ulva* species) in every growing season (from October to June) in each site were used to calculate the Euclidean distance among the sites, and then, cluster dendrograms for compositional similarities among the sites were developed by the Ward's method, which has a higher classification performance.

Overall differences in mean water temperature in every growing season among study sites of the Shimanto River were determined by one‐way analysis of variance (ANOVA). If the one‐way ANOVA for water temperature was significant, differences between study sites were assessed by Tukey's test. Similarly, statistical differences in mean salinity among sites were determined by Freedman's test, and then, Scheffé's test was used for pairwise comparison of the study sites.

Occurrence of asexual variants of *U. prolifera* along the salinity gradient of river water in the Shimanto River was assessed by logistic regression model. We assumed that the occurrence of asexual and sexual variants followed a binomial distribution and modeled the occurrence probability of the asexual form as a function of mean salinity in every growing season in each site. All the parameters of the logistic regression model were assessed with the maximum‐likelihood estimation method.

To assess the ratios between sporophyte and gametophyte and between male and female gametophytes in the general population, bootstrap simulation (Efron 1979) was conducted. We assumed that these ratios followed binomial distributions. Using 10,000 of the bootstrap samples generated, mean and standard deviation of these ratios were estimated by the maximum‐likelihood estimate. All the statistical analyses were performed using the program R v. 3.1.2 (R Core Team 2014).

## Results

### The Shimanto River

#### Growth pattern of *Ulva prolifera* and other species

**Figure 4:**
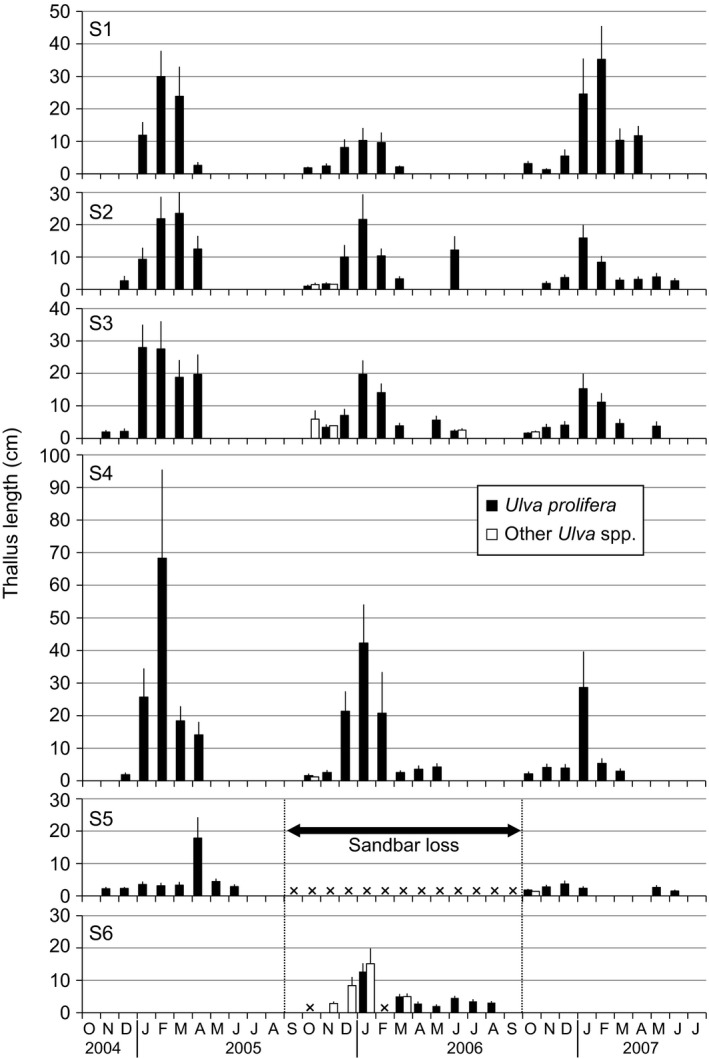
Seasonal change of thallus length average in *Ulva prolifera* and the other *Ulva* spp. at S1‐6. Bars are means with standard deviation. ×, no data at S5 due to the sandbar loss and at S6 due to heavy sea conditions with high waves. Each sample sizes are indicated in Fig. 5.

A total of 5,387 intact *Uva* thalli >1 cm were collected in the Shimanto River estuary between October 2004 and July 2007. In total, 5,152 thalli (96%) were identical to *U. prolifera* in microscopic morphology of thallus tissue, displaying mainly one pyrenoid per cell and chloroplasts filled the cell in surface view. The remnant of 235 thalli had a different microscopic morphology of having more than 2 pyrenoids per cell and/or the chloroplast tilted toward one side of the cell. Because the next generation cultured from their zoids had the same morphology again, they were identified as a different species from *U. prolifera*. Figure 4 shows the seasonal change of thallus length in *U. prolifera* and the other *Ulva* spp. in the Shimanto River estuary. Juvenile thalli of *U. prolifera* occurred from October to November, reaching the maximum length in January to February. They declined from February or March and disappeared by July. This seasonal pattern was similar at S1‐4 in the inner estuary from year to year, although the maximum value varied for each site and each year. However, such a seasonal pattern was ambiguous at S5 and S6 in the river mouth. The other *Ulva* spp. were not found at all before the loss of the sandbar due to the typhoon, but after that they appeared from the river mouth to the central estuarine site of S2. The maximum value of thallus length average in the other *Ulva* spp. was <6 cm in the inner estuary, while that in *U. prolifera* exceeded 10 cm at all inner sites every year. After the sandbar was re‐established, a low number of the other *Ulva* spp. occurred at S3 and S5 on October 2006, but later were not found again at any sites.

#### Frequency of species and reproductive modes

**Figure 5:**
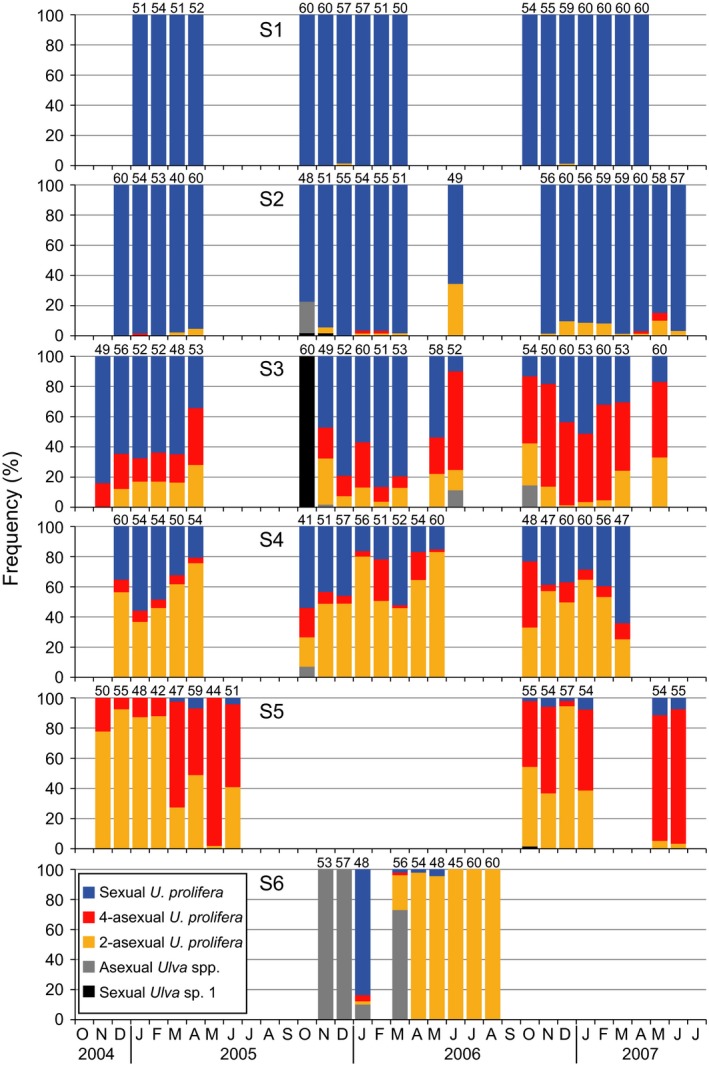
Seasonal change in frequency of sexual and asexual variants of *Ulva prolifera* and the other *Ulva* spp. at S1‐6. Sample sizes at each sampling are indicated above bars.

The other *Ulva* spp. were separated into one sexual species and more than two asexual species by culture experiments. The sexual species was thin and branching in gross morphology, being similar to *U. prolifera*. However, they had one to three, mostly two pyrenoids per cell different from that of *U. prolifera*. The sexual population at S3 on October 2005 included 56 sporophytes, two male and two female gametophytes. In crossing tests, their gametes failed to copulate with gametes of the identified *U. prolifera*, demonstrating a reproductive boundary between them. This unidentified sexual species was described as *Ulva* sp. 1 below. The other asexual species included multiple different types of microscopic morphology and life history, and these types were treated as an asexual species complex, because species separation of the asexuals was not possible by crossing tests, and an ongoing molecular analysis of this complex for the separation has not yet been completed. Seasonal frequency fluctuations in asexual and sexual variants of *U. prolifera*, sexual *Ulva* sp. 1, and the other asexual species complex in the Shimanto River estuary are shown in Figure 5. In *U. prolifera* the sexual variants clearly predominated from the center to upper estuary at S1 and S2, while the asexual variants conversely did at S5 in the lower margin. The mixed ratios of sexuals and asexuals were found in the intermediate sites of S3 and S4. This sexual/asexual distribution pattern was persistently observed over three consecutive growing seasons. In the asexuals, biflagellate type was more than the quadriflagellate type in number of individuals in almost all collections at S4, while conversely quadriflagellate type was more than biflagellate type at S3. A rapid succession of species dominance was observed at S6 where newly emerged after the sandbar loss. At first the asexual other *Ulva* spp. settled there, in the next, sexual *U. prolifera* occurred mixed with them for a short period only; finally, the asexual *U. prolifera* persistently occurred at 100% until the site of S6 disappeared by the sandbar re‐establishment.

### The Niyodo and Monobe rivers

**Figure 6:**
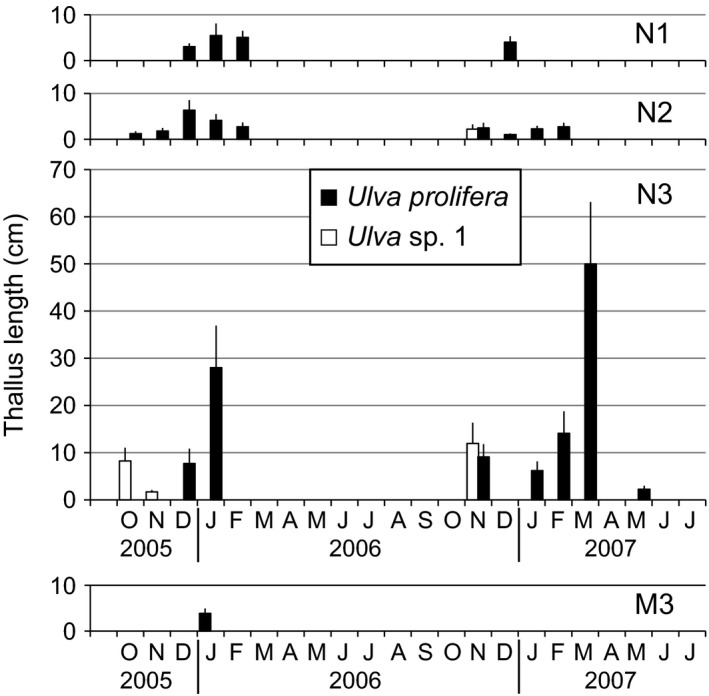
Seasonal change of thallus length average in *Ulva prolifera* and *Ulva* sp. 1 at N1‐3 in the Niyodo River and M3 in the Monobe River. Bars are means with standard deviation. Each sample sizes are indicated in Fig. 7.

Total 1,173 *Ulva* thalli were collected at three sites in the Niyodo River estuary for two growing seasons. Although 1,038 of them were consistent with *U. prolifera* from the Shimanto in microscopic morphology, the remaining 135 thalli (12%) had the same morphology of *Ulva* sp. 1 found in the Shimanto River. Their male and female gametes crossed with those from the Shimanto River. Therefore, they were regarded as the same species, *Ulva* sp. 1. No other *Ulva* species was found in the collected samples. Figure 6 shows the seasonal changes of thallus length in *U. prolifera* and *Ulva* sp. 1 in the estuaries of the Niyodo River (N1‐3) and Monobe River (M3). *Ulva* sp. 1 appeared at N2 and N3 during a short period in October and November, being consistent with the growing period in the Shimanto River. Although this species appeared in the inner estuary in the Shimanto River only during the period of sandbar loss, they repeatedly occurred in two consecutive years in the Niyodo River, which did not experience such a large topographical change. *Ulva* sp. 1 growing at N3 in the Niyodo River was larger in length than that in the Shimanto River. *Ulva prolifera* occurred from October to February at N1 and N2 in the inner estuary, having <10 cm thallus length. At N3 of the river mouth, growth and disappearance in *U. prolifera* rapidly happened, showing no stable seasonal growth pattern. In the Monobe River estuary, an *Ulva* population appeared only once at M3 through the 22 monthly field observations. All of the collected 54 *Ulva* thalli were microscopic‐morphologically identical with *U. prolifera* from the Shimanto and Niyodo rivers. The thallus length was only about 4 cm on average. No *Ulva* thallus was observed at the upper sites of M1 and M2.

**Figure 7:**
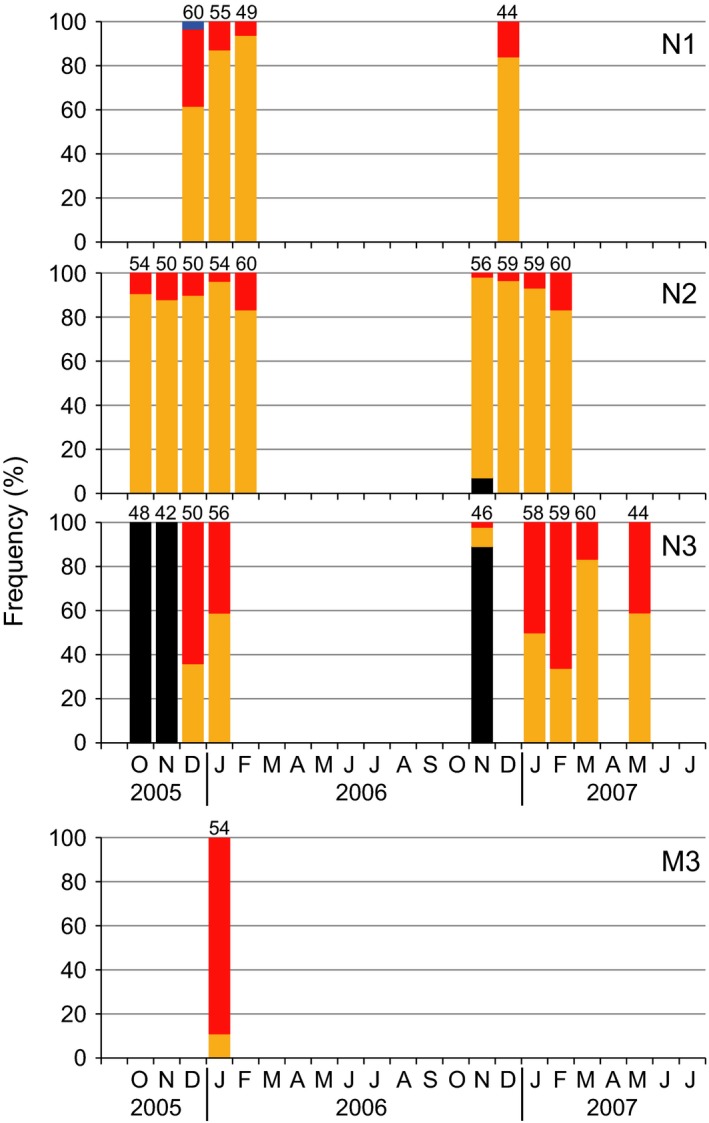
Seasonal change in frequency of sexual and asexual variants of *Ulva prolifera* and *Ulva* sp. 1 at N1‐3 in the Niyodo River and M3 in the Monobe River. Sample sizes at each sampling are indicated above bars. Bar colors indicate the same categories of species and life‐history type as shown in Fig. 5.

Figure 7 shows the seasonal change of frequency of asexual and sexual variants of *U. prolifera* and *Ulva* sp. 1 in the Niyodo and Monobe river estuaries. Except for two sexual individuals at N1 on December 2005, all *U. prolifera* thalli collected from the Niyodo River estuary were asexual variants. The two sexuals released gametes, of which both crossed with female gametes from the identified female gametophyte of *U. prolifera* from the Shimanto River. Therefore, they were identified as male gametophytes. Both biflagellate and quadriflagellate asexual types were found in every collection at all sites of the Niyodo River. The frequencies of biflagellate type at N1 and N2 in each growing season were more than 80%, while those at N3 dropped to approximately 50%. The small asexual population at M3 in the Monobe River was dominated with 89% by quadriflagellate type.

### Sexual/asexual ratios and salinity

**Figure 8:**
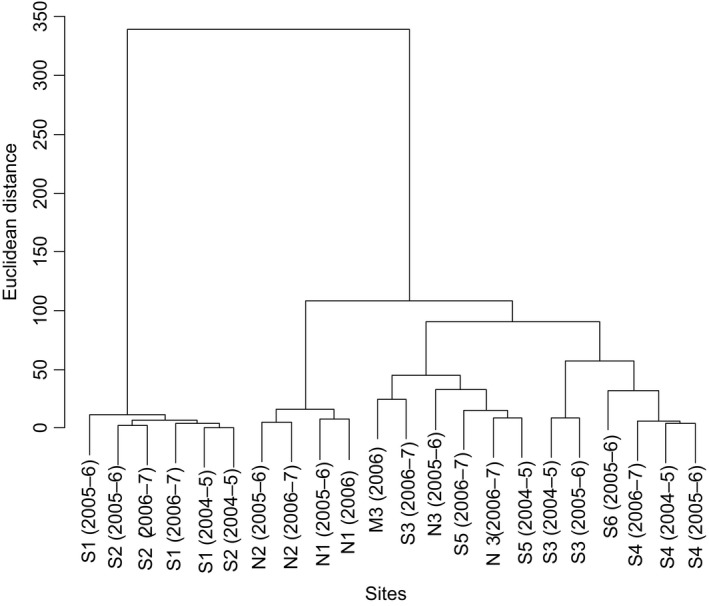
Compositional similarities in sexual and asexual variants of *Ulva prolifera* and other *Ulva* spp. among study sites in the estuaries of the Shimanto, Niyodo, and Monobe rivers. Numerals in parentheses indicate survey years.

Compositional similarities in sexual and asexual variants of *U. prolifera* and other *Ulva* species among the study sites are shown in Figure 8. The dendrogram was mainly divided into two groups: the sexual dominant group of S1 and S2 and the other group including asexual dominant or asexual–sexual mixed sites. Compositional similarity between S1 and S2 was higher, and annual variability of them was also smaller than the others. Next, the groups with a high asexual frequency divided into subgroups of N1, N2, and S4 with a small yearly variation and the remnant including N3 and S5 with the large variation. The sites with small yearly fluctuations were located in the inner estuaries, while ones with the large yearly fluctuations were at the seaward margins more exposed to the sea.

**Figure 9:**
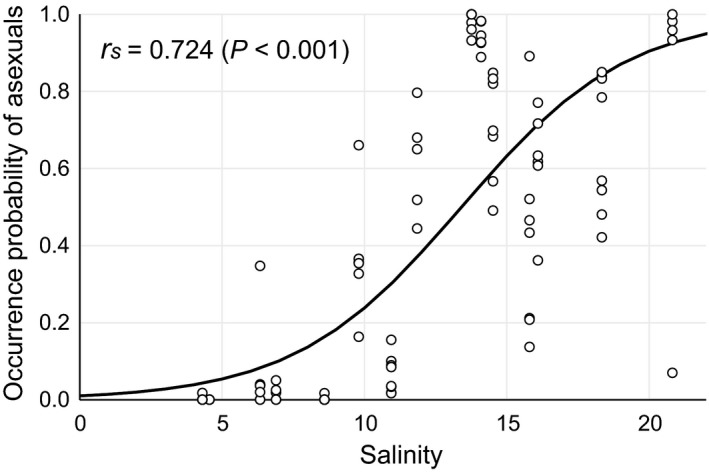
A relationship between monthly asexual rate and average salinity in every growth season at S1‐5. Regression line represents occurrence probability of asexual variant projected by the maximum estimation method. *r*_*s*_ denotes Spearman's correlation coefficient.

Correlation between asexual rate and salinities was analyzed from the data from the Shimanto River estuary where coexisting sexual and asexual variants were widely observed. Table 1 shows the averages of water temperature and salinity at around the lowest tide of each month for each growing season from October to June at S1‐6 and their statistically analyzed results. In the temperatures, comparatively lower values at the upstream sites of S1 and S2 were observed in the first and second growing seasons, but the average temperatures among all sites were not significantly different in the third season. On the other hand, although salinities had a large fluctuation due to a changeable freshwater discharge, the salinity averages of S1 and S2 were lower than ones of S3‐6 around the river mouth throughout the three growing seasons. In Figure 9, asexual rates were plotted to the salinity average in each growing season at each site of S1‐6. As a result, occurrence probability of asexual variants increased with the increase in river water salinity.

**Table 1:** Comparison of means of water temperature and salinity among the sites of S1‐6 in each growing season from October to June (*n* = 9). Values are means with standard deviation. Different letters on mean water temperature and mean salinity (a, b, c) indicate statistical differences at *P* < 0.05 within a given season

	Growing season	Site					
		S1	S2	S3	S4	S5	S6
Temperature	2004–2005	16.6^a^ ± 5.5	16.8^ab^ ± 4.6	18.0^ab^ ± 5.0	18.3^b^ ± 3.6	18.0^ab^ ± 3.6	
	2005–2006	16.6^ab^ ± 6.3	16.5^a^ ± 5.5	17.8^abc^ ± 5.4	18.8^c^ ± 5.4		18.0^bc^ ± 4.4
	2006–2007	18.2 ± 5.2	17.5 ± 4.9	18.2 ± 4.6	17.9 ± 4.3	17.9 ± 4.5	
Salinity	2004–2005	4.6^a^ ± 5.4	6.9^ab^ ± 5.0	9.8^ab^ ± 6.9	11.9^b^ ± 8.3	13.8^b^ ± 10.5	
	2005–2006	4.3^a^ ± 4.1	6.3^ab^ ± 5.0	15.8^abc^ ± 9.2	18.3^bc^ ± 9.3		20.8^c^ ± 8.9
	2006–2007	8.6^a^ ± 6.1	10.9^ab^ ± 5.7	14.5^b^ ± 7.2	16.1^b^ ± 7.1	14.1^ab^ ± 6.9	

### Phase frequency in sexual *Ulva prolifera*

**Figure 10:**
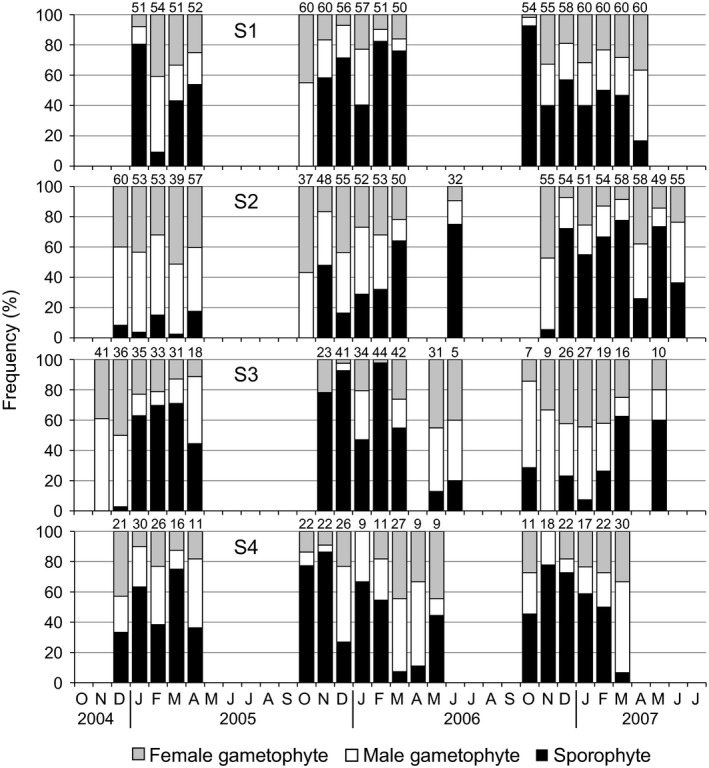
Seasonal change in frequency of male and female gametophytes and sporophytes of *Ulva prolifera* at the inner estuarine sites (S1‐4) in the Shimanto River. Sample sizes at each sampling are indicated above bars.

Figure 10 shows the seasonal fluctuation of frequency of male and female gametophytes and sporophytes in sexual *U. prolifera* at S1‐4, where the sexuals constantly occurred in all monthly samples. Monthly sporophyte ratios at each site rapidly changed, and no regular seasonal pattern was recognized. In the shortest case, the alternation of phase dominance happened at an interval of 1 month. The yearly sporophyte ratios at S2 and S3 were significantly different (Fisher's exact test, *P* < 0.05 and Tukey's test, *P* < 0.05). Gametophyte or sporophyte dominance changed yearly at the two sites. On the other hand, the yearly ratios at S1 and S4 have no significant difference between growing seasons (Fisher's exact test, *P* > 0.05). The yearly sporophyte percentages were kept at approximately 50% from year to year. Totally, in order to estimate the sporophyte ratio in the general population of *U. prolifera* in the Shimanto River estuary, the maximum‐likelihood estimate was simulated by a bootstrap method and was consequently 0.44 ± 0.03 (*n* = 10,000). In total 76 samples monthly collected, the ratios of male and female gametophytes in only 4 samples statistically differed from 1:1 (*χ* ^2^ test, *P* < 0.05). Female and male skewed ratios were observed in 1 and 3 samples, respectively. The maximum‐likelihood estimate of male ratio in the population was 0.53 ± 0.01 (*n* = 10,000).

## Discussion

### Invasive potential of other *Ulva* spp. into estuaries

Many ecological researches of *Ulva* (as syn. *Enteromorpha*) populations have been conducted without detailed species identification (e.g., Schories et al. 2000; Martins et al. 2001; Villares and Carballeira 2004), because they are morphologically similar that it can be very difficult to separate the forms from each other (Blomster et al. 2000). However, *Ulva* thalli are often found as mixed‐species populations, in which two or more species can sympatrically grow (Pregnall and Rudy 1985; Blomster et al. 2000). A recent molecular phylogenetic study has demonstrated that more than 10 *Ulva* species including *U. prolifera* occur with different seasonalities and various spatial distributions in small brackish lakes (Ogawa et al. 2013). The present study also showed that multiple *Ulva* spp. occurred in a narrow range around the river mouth (Fig. 5). Interestingly, the accidental collapse and re‐establishment of the sandbar revealed an invasive potential of other *Ulva* spp. into the estuary other than *U. prolifera*. Only when the river mouth was completely open to the sea, the other *Ulva* spp. colonized within the estuary. Particularly, *Ulva* sp. 1 consecutively occurring at the Niyodo River mouth (Fig. 7) displayed a clear pattern of appearance during the period of the sandbar absence and disappearance in its presence within the Shimanto River estuary (Fig. 5). These observations suggest that topographical change of river mouths would be an important factor to drive the species composition within the estuary. At the same period of 2006–2007, Sakaguchi et al. (2009) investigated the impacts of collapse and re‐establishment of the sandbar against the distributions and the densities of the copepod community in the Shimanto River estuary. They described that the sandbar disturbed the distribution pattern of some stenohaline copepods which subsequently did not enter the estuary from the sea. Stenohaline organisms are defined as being marine, only occurring in the mouths of estuaries, at salinities down to 25 (McLusky and Elliott 2004). *Ulva* sp. 1 in the present study can be classified into this ecological category. The river mouth topography seems especially to have a large effect on the migration of such stenohaline organisms. The larger temporal variations in species composition observed at the seaward margin than in the inner estuary (Fig. 8) would be caused by the highly invasive potential from the sea and the changeable topography of river mouth.

### Predominance of *Ulva prolifera* within estuary and its reproductive structure

Species number of macroalgae linearly decreases with the salinity decrease from fully marine to oligohaline conditions (Schubert et al. 2011). A paucity of species with an ability to tolerate a wide range of salinities is able to have abundant populations within the estuaries (McLusky and Elliott 2004). In accordance with the general tendency of species diversity in estuaries, only a single species *U. prolifera* abundantly predominated within the estuaries despite the invasive potential of multiple other *Ulva* spp. from the sea (Fig. 4). In the largest *U. prolifera* population, the sexual variants persistently occupied over a wide range from center to upstream end of the habitat, while the asexual variants occurred in a narrow reach of the seaward marginal habitat. It has been documented that the sexual *U. prolifera* strain from the Shimanto River has a nearly 100% viability against freshwater exposure for a week, under which other marine *Ulva* spp. almost all die (Ichihara et al. 2013). The low salinity tolerance in the sexual *U. prolifera* would enable its wide distribution within the estuary. Considering that the sexuals seldom or never grew in narrow estuaries such as the Niyodo and Monobe rivers (Fig. 7) and were competitively replaced by their asexuals or other species at seaward margin of the estuary as observed at S5 (Fig. 5), they can be definitely classified as true estuarine organism. This ecological category is featured by living in the central parts of estuaries commonly at salinities of 5–18, most in which could live in the sea but are apparently absent from the sea probably due to competition from other organisms (McLusky and Elliott 2004). The present observations suggest a moderate oligohaline to mesohaline area would be needed for the stable occurrence of the sexual *U. prolifera* populations. By contrast, the asexual variants preferred a higher salinity of mesohaline to polyhaline, where they rapidly colonized by means of zoosporoids. Asexual *U. prolifera* populations in brackish environments along the Sea of Japan have been shown to include 14 different genotypes, of which the composition differed between the high and low brackish sites (Ogawa et al. 2014). Also in *U. linza* being phylogenetically close to *U. prolifera*, Innes (1987) reported that the asexual populations separated by only a few hundred meters were genetically differentiated and the asexual samples from areas of low salinity were genetically similar but distinct from adjacent high salinity areas. Similarly in the present study, composition of biflagellate and quadriflagellate asexual types differed between adjacent sites such as S3 and S4 in the Shimanto River and N2 and N3 in the Niyodo River. As the biflagellate type occurred at a higher frequency in the upper area than the quadriflagellate type, the former seems to prefer lower salinity than the latter. Thus, the reproductive structure of *U. prolifera* populations can be summarized: (1) In estuaries with available oligohaline to mesohaline reaches, a mixed population of sexual and asexual variants occurs, forming a microgeographical distribution pattern of the sexuals in the inward and the asexuals in the seaward margin; (2) in estuaries without stable low saline reaches, only asexual variants occur as a smaller population for a shorter period; and (3) the asexual populations are composed by multiple ecotypes that are genetically differentiated. If this reproductive structure is a general trend in *U. prolifera* populations, it can be predicted that the asexual populations would be more commonly found than the sexual ones at macrogeographic scale, because river estuaries with low saline reaches available for the sexuals are limited. Indeed, there are few reports of sexual *U. prolifera* populations, while the exclusively asexual populations have been commonly found in the other brackish waters (Hiraoka et al. 2003; Ogawa et al. 2014). It has been proposed that macroscale geographic parthenogenesis is shaped by an environmental gradient which leads the sexuals and asexuals to different microenvironment preferences in dandelions (Verduijn et al. 2004). Likewise, the ecological differentiation between the sexuals and asexuals associated with saline environments may build up a macrogeographic distribution pattern in *U. prolifera*.

The asexual dominance of several marine macroalgae in the lower salinity regions has been explained by the impairment of the sexual reproductive system due to reduced performance of gametes or unsuccessful fertilization (Raven 1999; Serrão et al. 1999). However, our study in contrast demonstrates the persistent predominance of sexuals in the lower salinity reaches than the asexuals. The sexual variants showed drastic fluctuations of gametophyte/sporophyte ratios and constant 1:1 sex ratios of the gametophytes regardless of sites and seasons (Fig. 10). These observations indicate a frequent alternating of dioecious gametophytes and sporophytes through zygotic conjugation and meiotic spore production, suggesting that the sexual reproductive process would be robustly performed in the low salinity waters. In conclusion, the impairment of sexual processes by low salinity cannot explain the sexual–asexual distribution pattern in *U. prolifera*.

### An evolutionary explanation for the sexual–asexual distribution pattern

**Figure 11:**
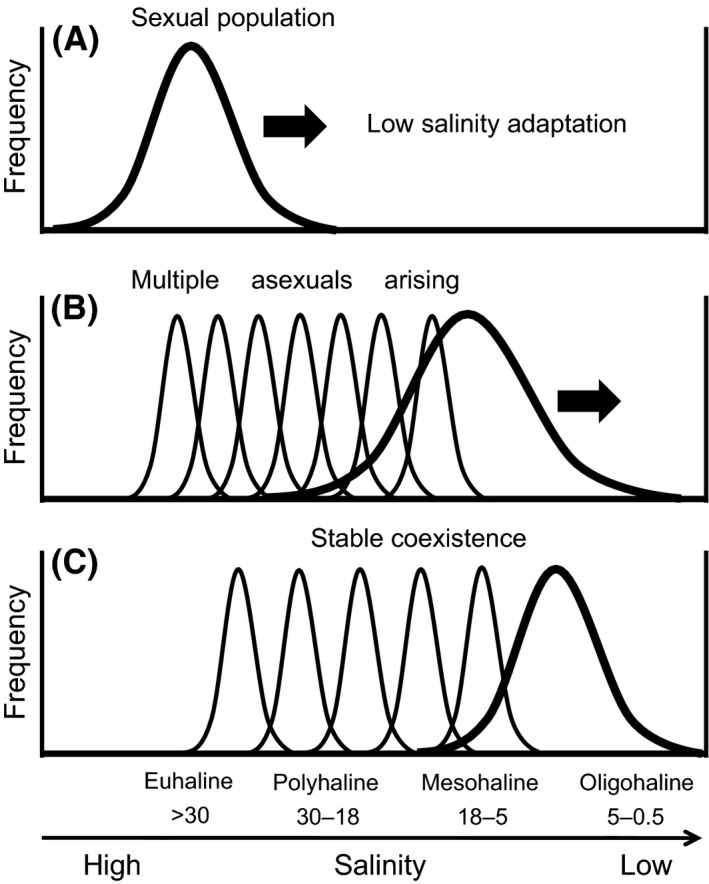
The evolutionary process of the sexual–asexual distribution pattern of brackish *Ulva prolifera* based on the FNV model. (A) A sexual population that separated from the marine *U. linza* population started to adapt to lower saline waters; (B) multiple asexuals arising from genetically variable sexual ancestors provided frozen genotypic variation on the low salinity adaptation; (C) selection eliminates asexuals that significantly overlap the niches of established asexuals and the sexual progenitors, and fixes an array of specialized asexuals that efficiently occupy the habitats.

The sexual–asexual distribution pattern in the true estuarine species was different from both B and C in Figure 2 that could be expected from patterns previously reported in marine organisms. Thus, our initial hypothesis was rejected. However, in a broad sense this pattern seems to be included in the asexual occurrences in marginal habitats, although asexuals of *U. prolifera* did not predominate in the upstream margin. This asymmetric occurrence by asexuals can be explained by its speciation process and the frozen niche variation (FNV) model. Although several hypotheses for the asexual occurrence in marginal habitats have been proposed (Haag and Ebert 2004; Kawecki 2008), the FNV model is supported by experimental data and population genetics (Hörandl 2009; Von Saltzwedel et al. 2014). The FNV model explains the asexual marginal occupation by niche partitioning between sexual ancestors and their hybrid apomictic progeny, the latter of which have a fixed subset of genetic variations from the sexuals (Vrijenhoek and Parker 2009). According to analysis of a large number of studies of vascular plant species, the ultimately successful asexual plants were concluded to be better regarded as a sign of sexual failure than as an indication of clonal success (Silvertown 2008). Similarly, it was recently proposed by molecular phylogeographic analyses in rockcress that the hybrid apomicts should be recognized to be trapped in the ecological niches of their sexual ancestors, supporting the FNV hypothesis (Mau et al. 2015). Molecular phylogeographic analyses (Shimada et al. 2008) and a hybridization study (Hiraoka et al. 2011) revealed that *U. prolifera* adapting to low salinity had recently speciated from marine *U. linza*. As the asexuals of *U. prolifera* being highly heterozygous with a high genotypic diversity (Ogawa et al. 2014), they would be repeatedly created from the sexual population which was undergoing adaptation to lower salinity. Based on the FNV model (cf. fig. 6.2 in Vrijenhoek and Parker 2009), we can concisely explain the evolutionary process of the sexual–asexual distribution pattern of *U. prolifera* as illustrated in Figure 11. This scenario explains why the asexuals do not occupy in the upstream margin, implying that any specialized asexuals which threaten the oligohaline to mesohaline habitats of the sexuals have not arisen yet; therefore, the stable coexistence of sexuals and asexuals is maintained at present. In the ancestral species *U. linza*, sexual populations are rare in Japan (Hiraoka et al. 2011), and only obligate asexual variants have been found from the well‐studied European and North American populations (Bliding 1963; Koeman 1985; Innes 1987). We infer this asexual dominance as a final result after the asexuals won in the competition with their sexuals. If any asexuals of *U. prolifera* with much lower saline tolerance arise in the future, they may also overwhelm their sexuals like in *U. linza*.

## Conflict of Interest

None declared.

## Supporting information


**Figure S1.** A sexual and two types of asexual life histories in *Ulva prolifera* found in river estuaries on Shikoku Island, Japan (cf. Hiraoka et al. 2003).
