# Supplementary figures and images for: Novel distribution pattern between coexisting sexual and obligate asexual variants of the true estuarine macroalga Ulva prolifera
Source: Ecol Evol. 2016 Apr 27;6(11):3658–71. doi: 10.1002/ece3.2149 (PMC5513300; doi:10.1002/ece3.2149)

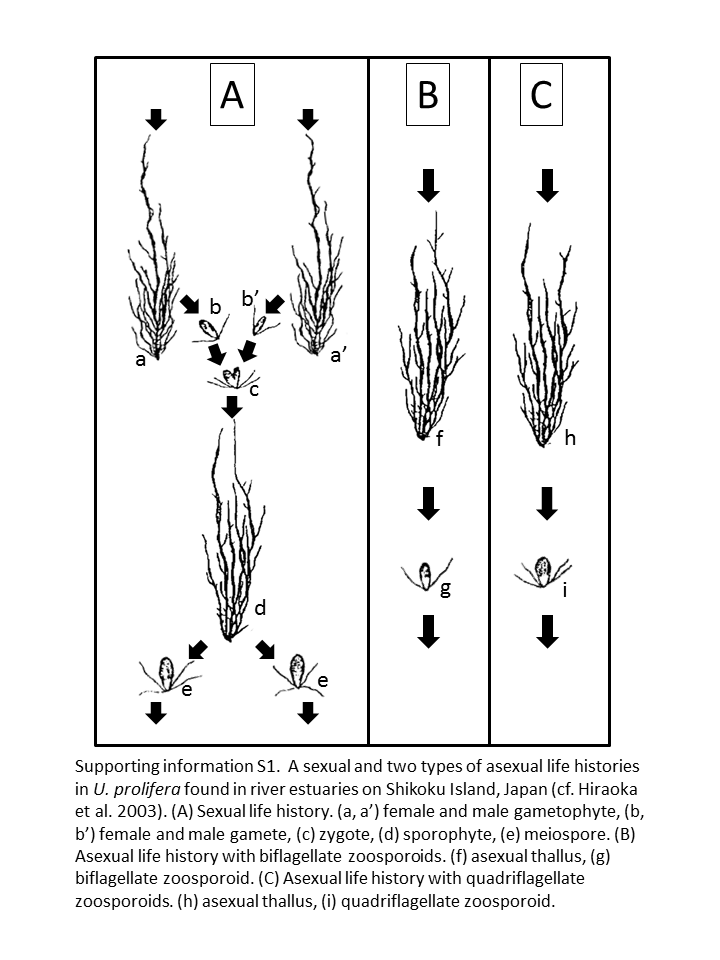

Supplement: Supplementary file 1 — Figure S1. A sexual and two types of asexual life histories in Ulva prolifera found in river estuaries on Shikoku Island, Japan (cf. Hiraoka et al. 2003). [file ECE3-6-3658-s001.tif]
